# Reducing radiation exposure and cancer risk for children with scoliosis: EOS the new gold standard

**DOI:** 10.1007/s43390-023-00653-6

**Published:** 2023-03-22

**Authors:** L. D. Rose, R. Williams, B. Ajayi, M. Abdalla, J. Bernard, T. Bishop, N. Papadakos, D. F. Lui

**Affiliations:** Department of Trauma and Orthopaedic Surgery, St George’s University Hospital, London, UK

**Keywords:** EOS, Radiation, Cancer, Risk, Children, Safety

## Abstract

**Purpose:**

Children are exposed to significant radiation doses during the investigation and treatment phases of scoliosis. EOS is a new form of low-dose radiation scan which also yields great image quality. However, currently its use is discouraged in the UK due to higher costs. We aimed to quantify the additional radiation dose and cancer risk.

**Methods:**

We retrospectively reviewed all paediatric cases who received both standing whole spine roentgenograms and EOS scans as part of their investigations for scoliosis during a six-month period. We compared the radiation doses between the two modalities and estimated the additional mean lifetime cancer risk per study.

**Results:**

We identified 206 children (mean age 14.4) who met the criteria of having both scans. Dose area products (dGycm^2^) were converted to estimated effective doses (mSv). The total mean doses were 0.68 mSv (PA 0.49 + Lat 0.19) for plain films, and 0.13 mSv (PA 0.08 + Lat 0.04) for EOS scans (*p* < 0.001). Additional lifetime cancer risk of a plain film was 543% greater than EOS for both sexes (1/10727 versus 1/5827 in males, 1/34483 versus 1/6350 in females).

**Conclusion:**

There is approximately 5.4-fold increase in risk of cancer for both boys and girls with roentgenograms over EOS, with girls being the most impacted. This carries a significant impact when considering the need for repeat imaging on additional lifetime malignancy risk in children. In our opinion, EOS dual planar scanning is the new gold standard when X-ray of the whole spine is required.

**Level of evidence:**

III.

## Introduction

An essential element in the investigation spinal deformity in children is plain two-dimensional imaging. This allows the surgeon to measure the magnitude and location of the curves, which is a key step in planning treatment for patients with scoliosis. Traditionally, standard roentgenograms have been used as the gold-standard for this purpose due to them being low-cost and readily available in most hospital settings [1]. Furthermore, most classifications and grading tools in orthopaedics are based on plain radiographs; such as the Cobb angle in scoliosis, or the C7 plumb-line for sagittal balance [2]. These are for example, a crucial part of the Lenke classification to guide treatment of scoliosis [3].

However, there is a significant concern amongst spinal surgeons about the amount of radiation we are exposing children to. Patients with spinal deformity are often subjected to multiple roentgenograms pre- and post-operatively or as part of their active monitoring process, with the cumulative effect of serial radiation exposures. As untreated Adolescent Idiopathic Scoliosis (AIS) is a low-mortality condition (unlike Early Onset Scoliosis), efforts to reduce iatrogenic morbidity and mortality from its investigation is paramount [4]. More recent high quality data have been able to confirm that children with scoliosis are at higher risk of cancers, and recommends the use of lower or radiation-free alternatives [5].

**Fig. 1:**
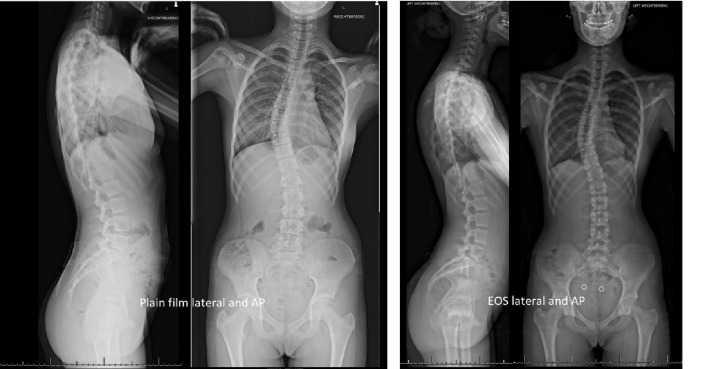
Plain whole spine roentgenogram (left) versus EOS whole spine (right) for qualitative image comparison

The advent of EOS bi-plane x-ray imaging has offered clinicians the opportunity to image the whole skeleton in the anatomical standing position with automatic image-stitching of simultaneous orthogonal collimated beam views, and with a smaller radiation dose than standard spine roentgenograms. It also has the advantage of being able to produce three-dimensional reconstructions, and is considered technically superior to plain roentgenograms (Fig. 1) on account that the use of image-stitching reduced the inaccuracies of the parabola effect. This same technique is responsible for the lower radiation dose [6, 7].

However, current NICE guidelines do not recommend EOS scans over x-rays citing: “Current evidence shows there are some patient benefits for people with spinal deformities in terms of radiation dose reduction and increased throughput. However, those benefits alone are insufficient to justify the cost of the system.” It continues to detail how there is a lack of evidence to quantify the benefits of image quality and standing whole spine view capabilities [8, 9].

The counter argument for this would be that equipment in the National Health Service is a depreciating asset, and requires regular updates to its assets to achieve reliability and better safety profiles. An investigative article in the Independent in 2021 found that hospitals were using very old technology, with St George’s University Hospital reportedly using a 44 year-old x-ray machine [10]. This would suggest that replacement through investment is imminent, and an opportunity to update equipment rather than replace like-for-like is present.

Alternatives to plain films, such as standing magnetic resonance imaging (MRI) are possible, and expose the patient to no additional radiation. However these have limitations for assessment of scoliosis compared to traditional radiographs and EOS. The chief disadvantages being speed, cost and availability. This mixed with coronal slices which make Cobb angle measurements more difficult and not all braces being compatible for “in brace” assessment are probably responsible for MRI not being a widely used modality for this purpose.

Our objective was to compare the radiation doses between the two imaging modalities and represent this as a malignancy risk per study. This would allow surgeons and patients to make a more informed decision when determining which image modality they wish to use. It may also lend weight to revising the NICE guidelines with respect to their discouragement of EOS scans.

## Methods

We retrospectively reviewed a cohort of all paediatric patients (under 18 years) who underwent both EOS scans and plain roentgenograms of standing whole spines whilst undergoing investigation for spinal deformity in a tertiary spinal centre in the UK*.* All patients were collected from September 2020 to March 2021.

We collected the individual projection dose area products (DAP) in dGy.cm^2^ for the combined postero-anterior (PA) and lateral projections from the hospital PACS database (or AP projections for patients who could not tolerate PA films). We then converted this to an estimated effective radiation dose, expressed as millisieverts (mSv) using a conversion coefficient for spinal radiographs using the ‘thoracic spine’ option, as this was perceived to be the closest to ‘whole spine’ [11].

We aimed to compare the average radiation dose of PA and lateral projections between the two modalities. We then utilised a validated lifetime risk of cancer calculator based on population radiation exposure following during events such as Hiroshima and Nagasaki nuclear fallouts to estimate the additional mean risk per study [11–13]. The ‘upper spine’ selection was perceived to be the most suitable in the absence of a ‘whole spine’ option, which may have underestimated the total risk, but proportionally should be appropriate for comparison. We subsequently compared the differences in lifetime cancer risk per study between EOS and plain films in our groups of males and females.

Statistical analysis using the paired *t* test on GraphPad software was used to compare the significance of the difference in doses between x-ray and EOS (using dose area product, which is the base data for our calculations), setting the significance level as *p* < 0.05.

## Results

We identified 206 children (mean age 14.4) who met the criteria of having both scans during the relevant time period. There were 19 males with a mean age 13.97 (range 1–17), and 187 females with a mean age of 14.5 (range 1–18). No further demographic data were collected.

Mean dose area products (DAP) were 21.4 dGycm^2^ (range 2.3–114.5) for lateral projection plain spine roentgenograms, and 20.4 dGycm^2^ (range 1.2–181.8) for PA projections. For EOS the DAP was 4.7 dGycm^2^ (range 1.8–13.4) for lateral projections and 3.5 dGycm^2^ (range 1.0–10.2) for PA projections.

Results of the paired *t* test indicated that there was a significant difference between radiation total DAP using plain radiographs (mean = 38.9, SD = 27.1) and EOS scans (mean = 7.9, SD = 3.3). *t* = 16.6, *p* < 0.001.

Dose area products (dGycm^2^) were converted to estimated effective doses (mSv) using published conversion coefficients [14]. The total mean doses were 0.68 mSv (PA 0.49 + Lat 0.19) for plain films, and 0.13 mSv (PA 0.08 + 0.04) for EOS scans (Table 1).

**Table 1:** Table showing estimated effective doses (EED) of AP and lateral projections for standard roentgenograms and EOS studies

Modality	PA EED (mean)	Lateral EED (mean)	Total EED (mean)
Plain films	0.49 mSv (0.03–4.36)	0.19 mSv (0.05–2.75)	0.68 mSv
EOS	0.08 mSv (0.02–0.24)	0.04 mSv (0.04–0.08)	0.13 mSv

For the males (mean age 14.0) the baseline lifetime cancer risk was 44.9%, with an additional 0.0093% increase after a single plain whole spine roentgenogram (1 in 10,272). After a single EOS scan this was an additional 0.0017% (1 in 58,275). The difference was 543% additional lifetime cancer risk using x-ray over EOS.

For the females (mean age 14.5) the baseline lifetime cancer risk was 37.5%, with an additional 0.0157% increase after a single plain whole spine roentgenogram (1 in 6350). After a single EOS scan, this was an additional 0.0029% (1 in 34,483) (Table 2). The difference was 543% additional lifetime cancer risk using x-ray over EOS.

**Table 2:** Table displaying the average increase in lifetime risk of developing cancer per study, based on gender and imaging modality

	Plain film	EOS	*p* value
Total dose (mSv)	0.68	0.13	< 0.001
Additional Male lifetime risk (%)	0.0093	0.0017	< 0.001
Additional Female lifetime risk (%)	0.0157	0.0029	< 0.001

## Discussion

From our data, we can see that plain films of the whole spine amount to much larger radiation doses than for the same study using EOS. The magnitude of difference of almost 5.5 is reflected in the lifetime cancer risk per study. Whilst males have a higher background risk, females receive a much larger increase in their additional risk per study.

For every radiograph we use instead of EOS, we are exposing a young girl to 0.0128% (0.0157 minus 0.0029) increased risk of developing a malignancy in her lifetime. Whilst that may seem small that means for every 10,000 plain radiographs of a whole spine 1.3 girls may develop cancer. When we consider that these girls may receive 16 scans in their lifetime that additional risk rises to 0.2% (1.3 in 625), assuming a linear correlation between dose exposure and risk. This means an additional 1 in 500 risk of developing a cancer in every girl treated for scoliosis in addition to their background risk. This could be reduced to 1 in 2750 if the practice became EOS scanning.

If one considers a standard chest radiograph to produce an effective dose of 0.14 mSv [15], and a daily background radiation dose as 0.0066 mSv [16], we can use this as a standard of measure. This would mean a standard plain film of a whole spine in a child would deliver 4.9 times the dose of radiation as one chest radiograph. This also would be equivalent to 103.68 days of background radiation.

Additionally, a single whole spine roentgenogram from our study is equivalent to 16.7 flights between London and New York (cosmic radiation doses 0.04 mSv per flight) [17].

Using an EOS scan for this same study in a child, the radiation dose would be 5.4 times less. Meaning only 0.9 chest radiographs, or 19.1 days of background radiation, or 3.1 long-haul flights. This also may be under-estimating the amount of chest radiographs, days of background radiation and long-haul flights as these items are based on adult estimated effective doses.

After 16 whole spine roentgenograms (average number of studies per patient in their life), the paediatric patient could receive 10.9 mSv of radiation, whilst the EOS patient receives only 2.02 mSv. Whilst we are aware that risk and radiation may not be in linear correlation, we use this as a surrogate to estimate overall risk by the end of the patient’s treatment.

In a clinical context, an increase in the prevalence of breast cancer amongst women with scoliosis is thought to be due to repeated exposure to medical x-rays as part of their monitoring and treatment of their spinal deformity in earlier life [18]. Whilst it is argued that the expected lifetime dose of repeated imaging is below the body’s tolerance levels (based on data following use of atomic weapons in the second world war), there is undeniably an increase in rates of cancer within scoliosis populations [9, 10]. Some suggest this could be attributable to the disease process of scoliosis itself. However, no randomised-controlled trial has ever been conducted to verify or refute this theory.

Furthermore, a more recent found that there was a 17-fold increase in the incidence of cancer (mostly breast and endometrial) amongst patients previously treated for adolescent idiopathic scoliosis, compared to the 0.3% prevalence in the general paediatric population. They found that on average patients were exposed to 16.3 radiographs at a mean dose of 1.6 mSv per study. Although interestingly, fertility was unaffected [19].

Ionising Radiation (Medical Exposure) Regulations (IR(ME)R) and the Royal College of Radiologists (UK) are very clear that clinicians and hospitals should keep the radiation dose “as low as reasonably practicable” when considering the investigation of choice. This leads us to consider the alternatives to plain films as an ethical and lawful duty for our patients [20]. This in conjunction with the results of our cohort, should encourage clinicians to review the potential benefits of utilising EOS more in future.

Our results are comparable to other studies on the standard EOS scan versus plain roentgenograms [21, 22]. A larger impact on radiation exposure and cancer risk is expected to be found with ‘ultra-low-dose’ EOS scanning, which requires further research.

We recognise the limitation in our methods of calculating risk could also be under-estimating the cancer risk, as the ‘thoracic spine’ tool was used instead of the unavailable option of ‘whole spine’. Combining all three is likely to over-estimate the risk, however.

We appreciate there are limitations in our conclusions, as equivalence or superiority between the two imaging modalities were outside of the scope of this study. To our knowledge, no validity studies exist to compare Cobb angle measurements between EOS and plain films. This requires further research.

In our trust, a standard whole spine roentgenogram carries a tariff of £150, compared to an £250 for a whole spine EOS biplanar study without analysis of coronal and sagittal parameters. We cannot comment on value with regards to cost-effectiveness with respect to QALY, however, we do not feel this cost difference is significant enough to be inhibitory to its use given the vast improvement in radiation reduction.

## Conclusion

Standard plain film imaging of the whole spine requires in excess of 5 times higher doses of radiation compared to dual planar EOS scans, which was statistically significant. This carries a significant impact when considering the need for repeat imaging on additional lifetime malignancy risk in children.

There is approximately 5.43-fold increase in risk of cancer with roentgenograms over EOS. However, young females are the most vulnerable to radiation, and these findings are validated by other literature on the same topic.

The use of EOS scans significantly reduces additional cancer risk, and on this basis we favour EOS dual planar for investigation of spinal deformity. We also suggest further studies to assess and quantify the image quality benefits over standard roentgenograms. This would be important in the review process for current NICE guidance. In our opinion, EOS dual planar scanning is the new gold standard when x-ray of the whole spine is required.

## Data Availability

Our data is freely available upon request from the corresponding author.
